# Cardiac Function in Fetuses with Congenital Diaphragmatic Hernia (CDH) Before and After Fetoscopic Endoluminal Tracheal Occlusion (FETO)—A Systematic Review

**DOI:** 10.3390/life16050813

**Published:** 2026-05-13

**Authors:** Emilia Piotrkowicz, Przemysław Kosiński

**Affiliations:** Department of Obstetrics, Perinatology, Gynecology and Reproductive Medicine, Medical University of Warsaw, 02-091 Warsaw, Poland

**Keywords:** congenital diaphragmatic hernia, fetoscopic endoluminal tracheal occlusion, cardiac function, perinatology

## Abstract

Congenital diaphragmatic hernia (CDH) is a severe developmental defect characterized by pulmonary hypoplasia, pulmonary hypertension, and cardiac dysfunction. Fetoscopic endoluminal tracheal occlusion (FETO) is a prenatal intervention used in severe CDH to stimulate lung growth, yet its effects on fetal cardiac function remain insufficiently understood. This systematic review aimed to evaluate the impact of FETO on cardiac structure and function in fetuses with CDH. A comprehensive literature search identified four studies that met the inclusion criteria. Most studies were retrospective and showed considerable heterogeneity in study design. Overall, the available evidence suggests that FETO does not negatively affect fetal cardiac function and may promote favorable cardiac remodeling. Several studies reported improved growth of left-sided cardiac structures and better right ventricular performance in FETO-treated fetuses compared with severity-matched controls. Given the limited number of studies and their methodological variability, these findings should be interpreted with caution. Further well-designed, prospective studies with standardized cardiac assessment protocols and clearly defined outcome measures are needed to better characterize the cardiovascular effects of FETO in CDH.

## 1. Introduction

### 1.1. Description of the Condition

Congenital diaphragmatic hernia (CDH) is a developmental defect of the fetal diaphragm that results in the herniation of abdominal organs into the thoracic cavity [1]. The estimated prevalence of CDH in Europe is 1 in 4000 live births [2]. Despite significant advances in surgical techniques and neonatal intensive care in recent years, mortality among liveborn infants with CDH remains high, reaching 30–50% [3].

The etiology of CDH remains largely unknown [4,5]. Most CDH cases occur sporadically, although a subset is genetically determined [6,7].

The majority (75–90%) of CDH cases are left-sided [8,9].

In isolated cases of CDH, the most widely used prognostic parameter is the lung-to-head circumference ratio (LHR). To account for gestational age, LHR is often expressed as an observed-to-expected (*o*/*e*) ratio [10]. Lower LHR values and reduced *o*/*e* ratios are associated with a poorer prognosis.

More than half of CDH cases are detected prenatally [8]. The condition is most commonly diagnosed between 22 and 24 weeks of gestation during the routine second-trimester ultrasound examination [1,9]. The diagnosis is typically based on characteristic sonographic findings, such as mediastinal shift and visualization of the stomach adjacent to or posterior to the fetal heart [1].

Congenital diaphragmatic hernia primarily affects the pulmonary, cardiovascular, and gastrointestinal systems of the fetus. The characteristic clinical triad of CDH consists of pulmonary hypoplasia, pulmonary hypertension, and ventricular dysfunction [1].

Cardiac performance is a key determinant of neonatal outcomes in patients with CDH. Early ventricular dysfunction has been associated with increased mortality and a greater need for extracorporeal membrane oxygenation (ECMO) after birth [11,12].

Currently, there are no widely accepted recommendations or guidelines for cardiac monitoring in fetuses with CDH. According to Kinsella et al. [13] and Patel et al. [14], the key principles guiding clinical management in CDH should include the following:

– Early and regular echocardiographic assessment of cardiac function and pulmonary arterial pressure;
– Targeted and individualized treatment based on clinical and echocardiographic findings;
– Screening for cardiac dysfunction, elevated pulmonary vascular resistance (PVR), and pulmonary hypoplasia in neonates born with CDH;
– Routine fetal evaluation of cardiac dimensions.

### 1.2. Management of CDH

Currently, two primary strategies are considered in the management of CDH. The first strategy, reserved typically for severe CDH, includes performing a prenatal fetoscopic endoluminal tracheal occlusion (FETO) procedure, followed by postnatal surgical repair of the diaphragmatic defect. The second strategy, reserved typically for mild cases, relies exclusively on postnatal surgical repair, often using a synthetic patch to close the hernia [15].

FETO is a procedure in which an inflatable balloon is endoscopically placed in the fetal trachea. Tracheal occlusion results in the retention of lung fluid, increasing intrapulmonary pressure and promoting lung growth [14]. Studies show that the FETO procedure yields the best outcomes when performed between 26 and 28 weeks of gestation in cases of severe congenital diaphragmatic hernia (CDH) and between 30 and 32 weeks in moderate cases [1]. The tracheal balloon is typically removed at 33–34 weeks of gestation to restore airway patency and allow for normal respiratory function at birth [16,17].

Performing the FETO procedure significantly improves survival in severe CDH cases, increasing rates to 24–50% [18,19]. The most common complications of FETO include premature rupture of membranes [18,19,20].

It is important to mention that performing the FETO procedure prenatally does not eliminate the need for postnatal surgical repair of the diaphragm defect, which is usually performed 24–48 h after birth [21,22,23]. The defect can be repaired via transthoracic or transabdominal rout, with use of minimally invasive techniques, (thoracoscopic or laparoscopic repair) [22,23,24].

### 1.3. Importance of This Review

Cardiac function is a key factor in evaluating the prognosis of patients with congenital diaphragmatic hernia (CDH). While numerous studies have compared the hearts of healthy fetuses with those affected by CDH, the effects of the fetal endoscopic tracheal occlusion (FETO) procedure on cardiac function remain less understood, with relatively few studies addressing this issue.

This systematic review aims to do the following:

1. Summarize and critically evaluate all available studies investigating the impact of FETO procedure on fetal cardiac function;
2. Assess the current state of knowledge regarding the impact of FETO on cardiac function in fetuses with CDH, both before and after birth;
3. Summarize the key alterations in cardiac morphology and function following the FETO procedure and evaluate their potential clinical significance;
4. Identify the limitations and gaps within the current body of evidence to outline potential directions for future investigation.

## 2. Review

### 2.1. Search Strategy and Study Selection

This review was conducted by searching the online databases of PubMed, Scopus, and Embase, without any restrictions on publication date. The most recent search for each database was performed on 4 March 2026. All studies of interest focused on the analysis of cardiac function in fetuses with CDH who underwent the FETO procedure. We included studies regardless of their primary objective, study design, control and study group characteristics, diagnostic modalities used, or timing of assessment. We excluded articles reporting exclusively on technical aspects and outcomes of the FETO procedure without evaluation of fetal cardiac function in CDH.

The literature search was conducted independently and simultaneously by two authors (E.P. and P.K.) and adapted to the specific technical requirements of each database. Only English-language keywords were used. The literature search was conducted using predefined keyword combinations related to cardiac structure, fetoscopic endoluminal tracheal occlusion (FETO), and congenital diaphragmatic hernia (CDH). The search strategy combined controlled vocabulary (MeSH in PubMed and Emtree in Embase) with free-text terms adapted to each database. Full database-specific search strategies are provided in the Table 1.

Full-text articles of the included studies were subsequently retrieved by the same two authors (E.P. and P.K.), with assistance from institutional librarians when necessary.

Text review and data extraction were conducted jointly by two authors (E.P. and P.K.) in a standardized manner. No disagreements arose during the study selection or data extraction process.

**Figure 1:**
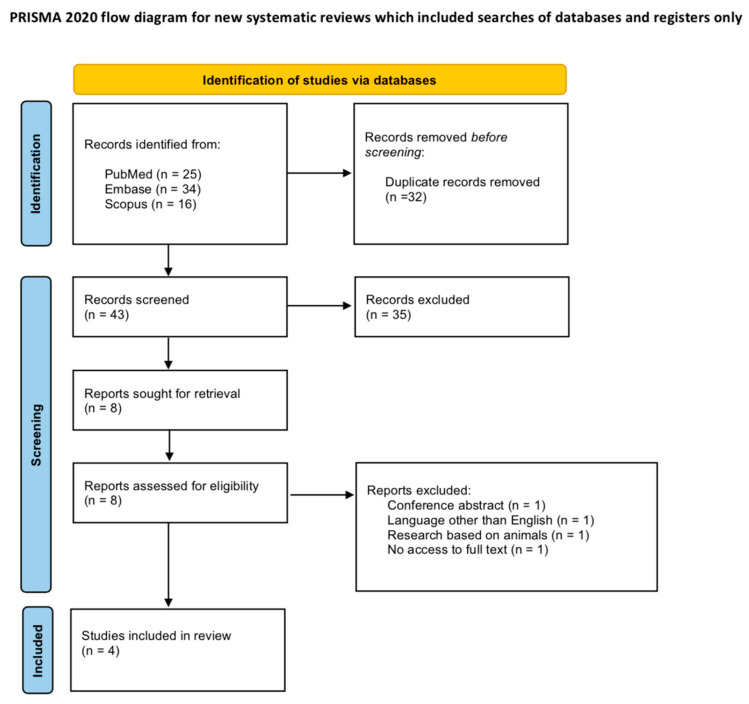
PRISMA flow chart of studies included in systematic review.

A total of 75 records were initially identified across the searched databases (PubMed = 25; Scopus = 16; Embase = 34). After manual removal of 32 duplicate records, the remaining studies were screened by title and abstract, resulting in the exclusion of 35 records that did not meet the predefined inclusion criteria. The full texts of the remaining reports were then assessed for eligibility. Four studies were subsequently excluded because they were conducted on animals, published in languages other than English, available only as conference abstracts, or lacked accessible full texts. Ultimately, four original studies were included in this systematic review and are presented in Table 2. The PRISMA flow chart of included studies is presented as Figure 1.

### 2.2. Study Characteristics

Of the four studies included in our systematic review, three used a retrospective design, and one was prospective.

The retrospective study by Rocha et al. [25] compared pre- and postnatal echocardiographic parameters in fetuses with CDH treated with FETO and in severity-matched controls who did not undergo the procedure. Prenatal cardiac measurements in the FETO group were obtained before the intervention, while postnatal parameters in both groups were collected prior to surgical CDH repair.

The study by Mieghem et al. [26] compared prenatal cardiac parameters in fetuses with CDH with those of a gestational age-matched healthy reference population. In fetuses undergoing FETO, additional echocardiographic assessments were performed 24 h before and after both the FETO-in and FETO-out procedures.

In the retrospective study by Dhillon et al. [27], left heart dimensions were assessed both prenatally and postnatally in fetuses with left-sided CDH treated with FETO and in severity-matched controls who did not undergo the procedure.

Finally, Avitabile et al. [28] compared cardiological outcomes between fetuses undergoing FETO and severity-matched controls whose parents declined the procedure as assessed at baseline, prior to surgical CDH repair, after repair, and at the last available follow-up. The key characteristics of control groups in all included studies are presented in Table 3.

### 2.3. Type of Participants

Inclusion criteria for the FETO procedure showed some variability across studies. Most commonly, eligible cases involved severe isolated left-sided CDH with significant pulmonary hypoplasia, defined using LHR or *o*/*e* LHR thresholds, often accompanied by intrathoracic liver herniation. Standard requirements also included normal karyotype, singleton pregnancy, maternal age ≥ 18 years, and a gestational age between 24 and 30 weeks. Severity thresholds of CDH varied, with *o*/*e* LHR cut-offs ranging from <26% to <30%, indicating a lack of uniformity in patient selection.

Exclusion criteria were less consistently reported. When specified, they generally involved maternal contraindications to anesthesia or surgery, preterm labor, associated fetal anomalies, or logistical barriers to follow-up. In several studies, no detailed exclusion criteria were provided beyond the failure to meet inclusion requirements.

### 2.4. Types of Intervention

FETO was the primary prenatal intervention evaluated across the included studies. However, the timing of both the FETO-in and FETO-out procedures varied between studies, which limits the direct comparability of results across the included research by introducing additional heterogeneity in treatment exposure.

Rocha et al. [26] performed FETO between 26 and 28 weeks, whereas Avitabile et al. [28] included cases of patients who underwent this procedure < 30 weeks of gestation. Mieghem et al. reported a median gestational age of 27 weeks for balloon insertion and 33 weeks for balloon removal. Dhillon et al. [27] included fetuses who underwent the FETO procedure between 26 and 29 weeks of gestation, with subsequent balloon retrieval performed between 32 and 35 weeks of gestation.

Control groups generally underwent expectant management, consisting of standard prenatal and postnatal care as described in Part 1.2. Management of CDH. None of the studies, however, specified the timing of postnatal diaphragmatic repair.

### 2.5. Outcome Measures

The studies demonstrated heterogeneous approaches to cardiac evaluation in fetuses with CDH undergoing FETO. Overall, left ventricular (LV) structure and function were the most frequently assessed parameters, reflecting the recognized impact of CDH on left heart development. Commonly analyzed variables included LV diameters and Z-scores, end-diastolic and end-systolic dimensions, volumes, and measures of systolic function such as shortening fraction, ejection fraction, and mitral annular plane systolic excursion (MAPSE). Some studies additionally evaluated diastolic function using Doppler indices (E/A ratios, isovolumetric relaxation, and contraction times).

Assessment of the right heart varied more widely. When reported, parameters included tricuspid and pulmonary valve dimensions, right ventricular major axis length, RV size and strain measurements, and tricuspid annular plane systolic excursion (TAPSE). Only two studies provided more detailed characterization of RV functional performance.

Additional cardiovascular markers reported in several studies included the main pulmonary artery and branch pulmonary artery diameters, cardiac axis, RV/LV systolic ratio, and global myocardial performance index (MPI/Tei index). Normalization methods differed: some used gestational age-specific Z-scores, while others applied observed-to-expected ratios, limiting direct comparison of results between studies.

Cardiac parameters assessed across studies are presented in Table 4.

## 3. Results

Each of the four included studies evaluated different cardiac parameters and focused on distinct outcome measures, resulting in substantial heterogeneity across the available evidence on cardiac remodeling following FETO.

Rocha et al. (2014) [26] demonstrated positive longitudinal growth of left-sided cardiac structures after FETO, including increases in aortic valve and left ventricular dimensions, with several postnatal parameters approaching normal ranges. Their findings suggest that FETO supports favorable remodeling of the left heart even before surgical repair.

In contrast, Dhillon et al. (2018) [27] found that left heart hypoplasia persisted after FETO, with meaningful improvement observed only following postnatal diaphragmatic repair. Notably, no correlations were identified between left-sided cardiac dimensions and fetal outcomes. After surgical correction, aortic valve dimensions remained smaller in the FETO group compared with controls, while mitral valve and left ventricular measurements did not differ significantly between the groups. These findings suggest that although FETO may help maintain prenatal hemodynamic stability, substantial structural recovery of the left heart largely takes place after birth. This conclusion stands in partial contrast to the more favorable prenatal remodeling reported by Rocha et al.

Mieghem et al. (2009) [26] demonstrated that the FETO procedure does not negatively impact fetal cardiac size or function. Instead, they reported a reduction in the myocardial performance index (MPI), suggesting an improvement in global cardiac performance. Importantly, both LV size and systolic function remained stable after balloon placement and were not adversely affected following balloon removal.

Finally, Avitabile et al. (2024) [28] demonstrated that FETO-treated infants had better RV function, less LV hypoplasia, and reduced ventricular septal displacement compared with non-FETO controls of similar severity. Although the findings are suggestive of favorable cardiac remodeling, the observed reduction in left ventricular shortening fraction indicates that functional parameters may not uniformly improve.

The detailed results and conclusions of each study are presented in Table 5.

## 4. Discussion

### 4.1. Summary of Results

This systematic review synthesizes all currently available evidence concerning the effects of FETO procedure on cardiac structure and function in fetuses with left-sided CDH. Although the number of published studies remains limited, several consistent trends emerge. Overall, FETO appears to promote favorable cardiac remodeling—without inducing measurable impairment in global cardiac performance. Among the included investigations, Rocha et al. and Avitabile et al. reported clear beneficial anatomical and functional changes following the procedure, while studies by Dhillon et al. and Mieghem et al. demonstrated no evidence of cardiac deterioration attributable to FETO.

A key finding across the included studies is the partial normalization of left-sided cardiac structures following FETO. Rocha et al. [25] reported that the intervention may promote improved growth of the left ventricle and aortic valve as well as closer-to-expected dimensions of the left pulmonary artery. Similarly, Avitabile et al. [28] demonstrated that FETO is associated with less severe left ventricular hypoplasia and reduced ventricular septal displacement. However, in contrast to those findings, Dhillon et al. [27] observed persistent left heart hypoplasia prenatally, with improvement only evident after postnatal intervention, indicating that the degree and timing of structural recovery may vary between individuals and requires longer-term follow-up.

An emerging insight from more recent work, particularly that of Avitabile et al. [28], is also the positive influence of FETO on right ventricular adaptation—improvements in RV dilation, septal position, and wall strain parameters. This perspective broadens the clinical significance of FETO, suggesting that its benefits extend beyond promoting lung growth to supporting more physiological cardiac development.

It is important to note that interpretation of results across studies should consider several potential sources of heterogeneity. These include differences in the timing of the FETO procedure and cardiac assessments, variability in echocardiographic techniques and evaluated parameters, and use of different normalization methods. Such methodological variability may partly explain the differences in reported cardiac outcomes.

#### Strengths and Limitations

Strengths of this review include the use of a comprehensive search strategy across multiple databases and strict adherence to PRISMA guidelines. To the best of our knowledge, the studies included represent all currently available evidence examining the impact of the FETO procedure on fetal cardiac function.

The review also benefits from the fact that most included studies used clearly defined inclusion and exclusion criteria, employed experienced examiners, and provided adequate descriptions of used echocardiographic techniques. Several studies performed inter- and intra-observer comparisons and applied appropriate statistical analyses, which strengthens the reliability of the extracted data.

Despite these strengths, several limitations should be acknowledged. The review shows considerable heterogeneity among the included studies, particularly regarding patient selection, timing of intervention, and the type of cardiac or clinical parameters assessed. Although inclusion criteria were generally well-described, they varied substantially across studies.

Additional limitations included the predominance of retrospective designs, small sample sizes, and non-standardized imaging protocols. Potential selection bias associated with referral-center cohorts further reduces the generalizability of the findings. The lack of standardized fetal cardiac assessment protocols further limits comparability between studies and highlights the need for uniform echocardiographic frameworks in future research. Finally, language restrictions may also have influenced the overall results. Taken together, these factors restrict the ability to perform a meta-analysis and highlight the need for future high-quality, prospective, and ideally randomized studies.

The strengths and limitations specific to each study are summarized in Table 6.

## 5. Conclusions

Despite limitations, current evidence suggests that FETO does not worsen cardiac performance and may provide structural and functional benefits in the perinatal period.

Given the strong association between cardiac dysfunction and mortality in CDH, these findings provide reassurance regarding the safety of the intervention and support its application in severe left-sided CDH, where potential gains in pulmonary development are crucial.

Based on the available evidence, several recommendations for future research can be proposed. First, there is a clear need to implement standardized fetal and postnatal echocardiographic assessment protocols, including uniform timing of examinations and consistent use of normalization methods to improve comparability across studies.

Second, future research should prioritize prospective, multicenter studies with larger patient cohorts to reduce selection bias and enhance the generalizability of findings.

Third, incorporation of advanced echocardiographic techniques, such as myocardial strain analysis and speckle tracking, is strongly recommended, as these methods may allow earlier and more sensitive detection of subtle cardiac dysfunction. These novel methods enable detailed evaluation of myocardial mechanics, including subtle changes in ventricular performance that may not be detected by standard measurements. Their broader implementation in future studies could improve the precision of cardiac assessment in fetuses with CDH and contribute to a more comprehensive understanding of the cardiovascular effects of the FETO procedure.

In addition, longitudinal follow-up of patients extending beyond the neonatal period should be routinely included to better understand the long-term cardiovascular consequences of FETO procedure.

Finally, closer integration of cardiac assessment into clinical decision-making algorithms for FETO eligibility and postnatal management may help optimize patient selection and improve overall outcomes.

## Figures and Tables

**Table 1:** Full search strategies.

Database	Search String
PubMed	((“heart” [MeSH Terms] OR “myocardium” [MeSH Terms] OR cardiac [Title/Abstract] OR hear t[Title/Abstract] OR myocardial [Title/Abstract] OR myocardium [Title/Abstract] OR ventricular [Title/Abstract] OR “heart ventricles” [Title/Abstract]) AND (“fetoscopic endoluminal tracheal occlusion” [Title/Abstract] OR FETO [Title/Abstract]) AND (“hernia, diaphragmatic, congenital” [MeSH Terms] OR “congenital diaphragmatic hernia” [Title/Abstract] OR CDH [Title/Abstract]))
Scopus	(TITLE-ABS-KEY (cardiac OR heart OR myocardial OR myocardium OR ventricular OR “heart ventricles”)) AND (TITLE-ABS-KEY (“fetoscopic endoluminal tracheal occlusion” OR FETO)) AND (TITLE-ABS-KEY (“congenital diaphragmatic hernia” OR CDH))
Embase	(‘heart’/exp OR ‘myocardium’/exp OR cardiac:ti,ab OR heart:ti,ab OR myocardial:ti,ab OR myocardium:ti,ab OR ventricular:ti,ab OR “heart ventricle”:ti,ab OR ventricl:ti,ab) AND (‘fetoscopic endoluminal tracheal occlusion’:ti,ab OR feto:ti,ab) AND (‘congenital diaphragmatic hernia’/exp OR “congenital diaphragmatic hernia”:ti,ab OR cdh:ti,ab)

**Table 2:** Main characteristics of studies included in this review.

Article	Study Type	Population	Time of the Study
Rocha et al. (2014)	Retrospective, single-center, case-control study	34 patients (9 FETO, 25 controls)	June 2000–June 2010
Dhillon et al. (2018)	Retrospective, single-center, case-control study	30 patients (12 FETO, 18 controls)	January 2007–December 2016
Mieghem et al. (2009)	Prospective, single-center, cross-sectional study	22 patients (12 FETO, 10 controls)	1 July 2006–31 December 2007
Avitabile et al. (2024)	Retrospective, single-center, cohort study	32 patients (10 FETO, 22 control)	1 September 2016–1 November 2021

**Table 3:** Characteristics of control groups across the included studies.

Article	Control Group
Rocha et al. (2014) [25]	26 patients 11 declined the FETO procedure or were randomized to postnatal therapy in the setting of a randomized controlled trial; 15 were excluded for reasons not related to the anomaly.
Dhillon et al. (2018) [27]	18 patients (all were randomized to postnatal therapy in the setting of the randomized control trial).
Mieghem et al. (2009) [26]	10 patients (all patients did not meet the FETO inclusion criteria). Additionally, cardiac parameters from all 27 fetuses with CDH included in the study were compared with those of 117 healthy fetuses from the authors’ previous study.
Avitabile et al. (2024) [28]	22 patients (all were eligible for the procedure, but the parents declined).

**Table 4:** Cardiac parameters assessed across studies.

Article	Compared Cardiac Parameters					
	Heart Structure	Systolic Function	Diastolic Function	Heart Mechanics	Pulmonary Artery Metrics	Global Performance Indices
Rocha et al. (2014)	Mitral valve (MV) diameter Z-score Aortic valve (AoV) diameter Z-score Left ventricle (LV) major axis length Z-score Left ventricle short axis dimension (LVSAD) and area Tricuspid valve (TV) diameter Z-score Right ventricle (RV) major axis length Z-score	Left ventricle output	Left ventricle end-diastolic volume (LVEDV) indexed to BSA		Pulmonary valve (PV) diameter Z-score Main pulmonary artery diameter (MPA) Z-score Left and right branch pulmonary artery diameters (LPA, RPA) Z-scores	
Dhillon et al. (2018)	Mitral valve (MV) diameter Z-score Mitral valve size Aortic valve (AoV) diameter Z-score Aortic valve size Left ventricular short-axis dimension (LVSAD) Left ventricular long-axis dimension (LVLAD)	Aortic valve cardiac output	Left ventricular end-diastolic dimension (LVEDD) Mitral valve cardiac output			
Mieghem et al. (2009)	Cardiac axis	End-systolic diameter of the left ventricle (LVESD) LV global longitudinal strain (LVGLS) End-systolic LV eccentricity index (LVEI) Shortening fraction (SF) Ejection fraction (EF) Ejection time (ET)	Mitral E/A index End-diastolic diameter of the left ventricle (LVEDD)	Isovolumetric contraction time (ICR) Isovolumetric relaxation time (IRT)		Myocardial performance index (MPI, Tei index)
Avitabile et al. (2024)	Right ventricle (RV) size	Left ventricle (LV) M-mode internal dimension Z-scores in systole (LVIDSZ) RV global longitudinal strain (RVGLS) RV free wall strain (RVFWS) Tricuspid annular plane systolic excursion Z-score (TAPSEZ) RV/LV systolic ratio	Left ventricle (LV) M-mode internal dimension Z-scores in diastole (LVIDDZ)	LV shortening fraction RV fractional area change (RVFAC)		

**Table 5:** Results and conclusions of assessed studies.

Article	Statistically Significant Results	Conclusions
Rocha et al. (2014) [25]	Fetuses that underwent FETO experienced better subsequent/longitudinal growth of the AoV (compared parameters: fetal Z-score AoV, postnatal Z-score AoV, *p* < 0.005). Fetuses that underwent FETO experienced better subsequent/longitudinal growth of the LV length (compared parameters: fetal Z-score LV length, postnatal Z-score LV length, *p* < 0.005). In fetuses treated with FETO, postnatal LPA diameter and LV end-diastolic volume indexed to body surface area were significantly closer to normal values prior to surgical repair compared with the control group (*p* = 0.039). LV:RV ratio was larger in the FETO infants versus in the control infants at birth (*p* = 0.001).	The results of the study show a positive increase in sizes of left heart structures in neonates who had undergone FETO in comparison with a cohort who had CDH of similar severity but did not undergo prenatal interventions.
Dhillon et al. (2018) [27]	The fetal aortic valve and mitral valve Z-scores decreased from baseline in the FETO group after final intervention (*p* = 0.01 and *p* = 0.02). In the control group, left heart hypoplasia (LHH) progressed through gestation, with a significant decrease in the fetal left ventricle short-axis dimension (LVSAD) Z-score (*p* < 0.01). Within the FETO group, there was no significant change in postnatal left heart dimensions after CDH repair, while a significant increase in the control postnatal mitral valve (MV) after CDH repair was seen (*p* = 0.01). The aortic valve size was noted to be smaller in the FETO group following CDH repair when compared to controls (*p* = 0.04). On comparison of change in Z scores from the fetal to the postnatal period between groups, there was a greater decrease in left ventricular short-axis dimension from the first fetal echocardiogram to the pre-CDH repair echocardiogram in the control group (*p* = 0.02), while a larger drop in the aortic valve Z score was noted between first fetal echocardiogram and post-CDH repair in the FETO cohort (*p* < 0.01).	The study showed that left heart hypoplasia persisted following the FETO procedure but exhibited partial normalization after postnatal surgical repair of CDH. No associations were found between left heart dimensions and outcomes.
Mieghem et al. (2009) [26]	Following the FETO procedure, LV end-diastolic and end-systolic dimensions remained markedly reduced in CDH fetuses, measuring on average 32% and 37% smaller, respectively, than those of control fetuses (*p* < 0.0001). The study showed a significant displacement of the cardiac axis between study and control group (*p* < 0.0001). FETO reduced the myocardial performance index (MPI) in all but one patient (*p* = 0.004), but reversal had no significant effect on MPI. There was a shortening of the ICT from 29.6 ± 8.4 ms before FETO to 21.8 ± 7.0 ms after occlusion (*p* = 0.009).	The study reported no adverse effects on cardiac axis, left ventricular size, or cardiac function in fetuses with severe CDH who underwent FETO followed by in-utero balloon removal.
Avitabile et al. (2024) [28]	RV/LV ratio and LVEI were lower in FETO patients compared to control patients (*p* = 0.01). LV hypoplasia was less severe in FETO patients compared to control patients (*p* = 0.01). LV shortening fraction was lower in FETO patients (*p* = 0.02). In the post-operative period, FETO patients demonstrated better RV function compared to control patients as measured by RVFAC (*p* < 0.01), RVGLS (*p* = 0.02), and RVFWS (*p* = 0.05).	Patients who underwent FETO demonstrated less severe RV dilation, less ventricular septal displacement, and less severe LV hypoplasia on first echocardiogram compared to control patients of similar CDH severity. While RV function improved in both groups over time, improvement in RV dilation and septal position measured by LVEI were greater in FETO patients. FETO patients demonstrated smaller increases in LV dimensions from first to last echocardiogram compared to control patients. FETO patients demonstrated improved RV dilation and ventricular septal position but smaller increases in LV size compared to control patients of similar CDH severity.

**Table 6:** Strengths and limitations of compared studies analyzed with accordance to PICOTS framework.

Article	Strengths		Limitations	
Rocha et al. (2014) [25]	Population: well-defined inclusion criteria Comparison: adequate description of control group Outcomes: the compared cardiac parameters are well-described; cardiac measurements were independently reviewed by two observers Study design: the study types are well-described; statistical analyses are appropriately selected and clearly justified	Population: well-defined exclusion criteria Intervention: detailed information on the FETO procedure, including the timing of balloon placement and the technique used to perform the intervention	Population: small number of included patients; Differences in FETO inclusion and exclusion criteria between the studies Intervention: differences in the timing of FETO insertion and removal Comparison: differences in the criteria used to justify control group exclusion Outcomes: the compared cardiac parameters differ between studies Timing: the timing when the cardiac parameters were compared differs between studies Study design: all studies are designed as single-center studies	Population: imbalance in the number of participants between the study and control groups Intervention: no information about the timing of FETO-out procedure Outcomes: the cardiac parameters were analyzed using 2D echocardiography Timing: the timing of cardiac assessments was insufficiently specified Study design: the study is designed as a case-control study; inter-observer variability was considerable for certain parameter measurements
Dhillon et al. (2018) [27]		Intervention: detailed information on the FETO procedure performance and the technique used to perform the intervention		Population: no well-defined exclusion criteria. Patients with minor congenital heart defects were included in the study; among the patients in the study group, five were found to have genetic abnormalities of uncertain significance Comparison: 3 three individuals in the control group exhibited genetic variants of uncertain significance. Outcomes: pPostnatal ventricular function parameters were not included in the study;. prenatal cardiac Z-scores were calculated based on gestational age, while postnatal Z-scores were based on body surface area Study design: the study is designed as a case-control study; interobserver and intraobserver variability analyses were not performed
Mieghem et al. (2009) [26]				Population: no well-defined exclusion criteria;. iImbalance in the number of participants when cardiac parameters are compared between CDH patients (*n* = 27) and healthy control groups (*n* = 117). Intervention: lLimited details regarding the performance of the FETO procedure. Comparison: patients included in control group are not matched for CDH severity with the study group. Outcomes: iIt is unclear which prenatal echocardiogram was selected as the baseline for each patient, and whether the patients were comparable in terms of gestational age at the initial scan or if Z-scores were used to standardize the measurements; the cardiac parameters were analyzed using 2D echocardiography Study design: the study is designed as a cross-sectional study; the study was conducted at a referral center for fetal surgery; the study was more cross-sectional than longitudinal
Avitabile et al. (2024) [28]				Population: not all exclusion criteria are well-described; imbalance in the number of participants between the study and control groups Intervention: limited details regarding the performance of the FETO procedure Study design: the study is designed as a cohort study; the cross-sectional design does not allow to assess the effect of medical or surgical interventions on cardiac function

## Data Availability

Data sharing is not applicable to this article as no datasets were generated during this literature review. All data analyzed are included in this published article.
